# Supplementary material for: Ovule Development and in Planta Transformation of Paphiopedilum Maudiae by Agrobacterium-Mediated Ovary-Injection
Source: Int J Mol Sci. 2020 Dec 23;22(1):84. doi: 10.3390/ijms22010084 (PMC7795287; doi:10.3390/ijms22010084)
Supplement: Supplementary file 1 [file ijms-22-00084-s001.zip › ijms-1047367-for proofreading-supplementary/Supplemental information 1-3/Figure S1.docx]

**Supplemental information 1**


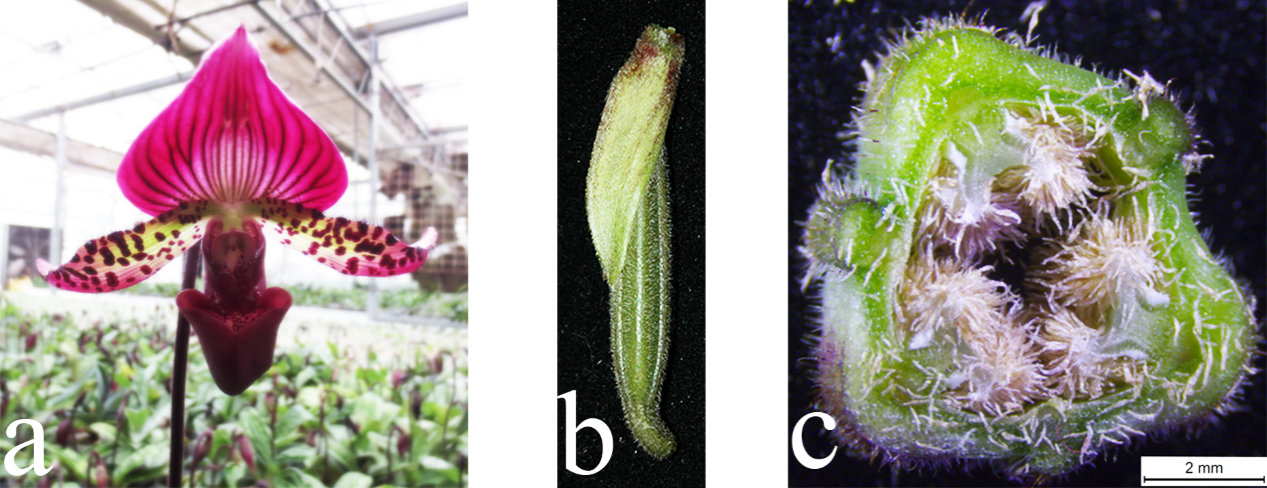


**Figure S1**. Flower and capsule of *Paphiopedilum* Maudiae. **a**: Flower; **b**: Capsule; **c**: Capsule cross section.
