# Supplementary material for: Ovule Development and in Planta Transformation of Paphiopedilum Maudiae by Agrobacterium-Mediated Ovary-Injection
Source: Int J Mol Sci. 2020 Dec 23;22(1):84. doi: 10.3390/ijms22010084 (PMC7795287; doi:10.3390/ijms22010084)
Supplement: Supplementary file 1 [file ijms-22-00084-s001.zip › ijms-1047367-for proofreading-supplementary/Supplemental information 1-3/Figure S2.docx]

**Supplemental information 2**

LB

Hyg（R）

RB

35S

GUS

CeFT

BamHⅠ

XbaⅠ

35S

Ubi

Nos polyA

35S polyA

Figure S2. Schematic diagram of T-DNA region of pUI1301/CeFT 35S, CaMV 35S promoter; Hygromycin (R), hygromycin resistant gene; 35S polyA, CaMV 35S terminator; GUS, β-glucosidase; Nos polyA, terminator of the Nopaline synthase gene; LB and RB represented the left border and right border of the T-DNA region, respectively.
