# Supplementary figures and images for: Ovule Development and in Planta Transformation of Paphiopedilum Maudiae by Agrobacterium-Mediated Ovary-Injection
Source: Int J Mol Sci. 2020 Dec 23;22(1):84. doi: 10.3390/ijms22010084 (PMC7795287; doi:10.3390/ijms22010084)

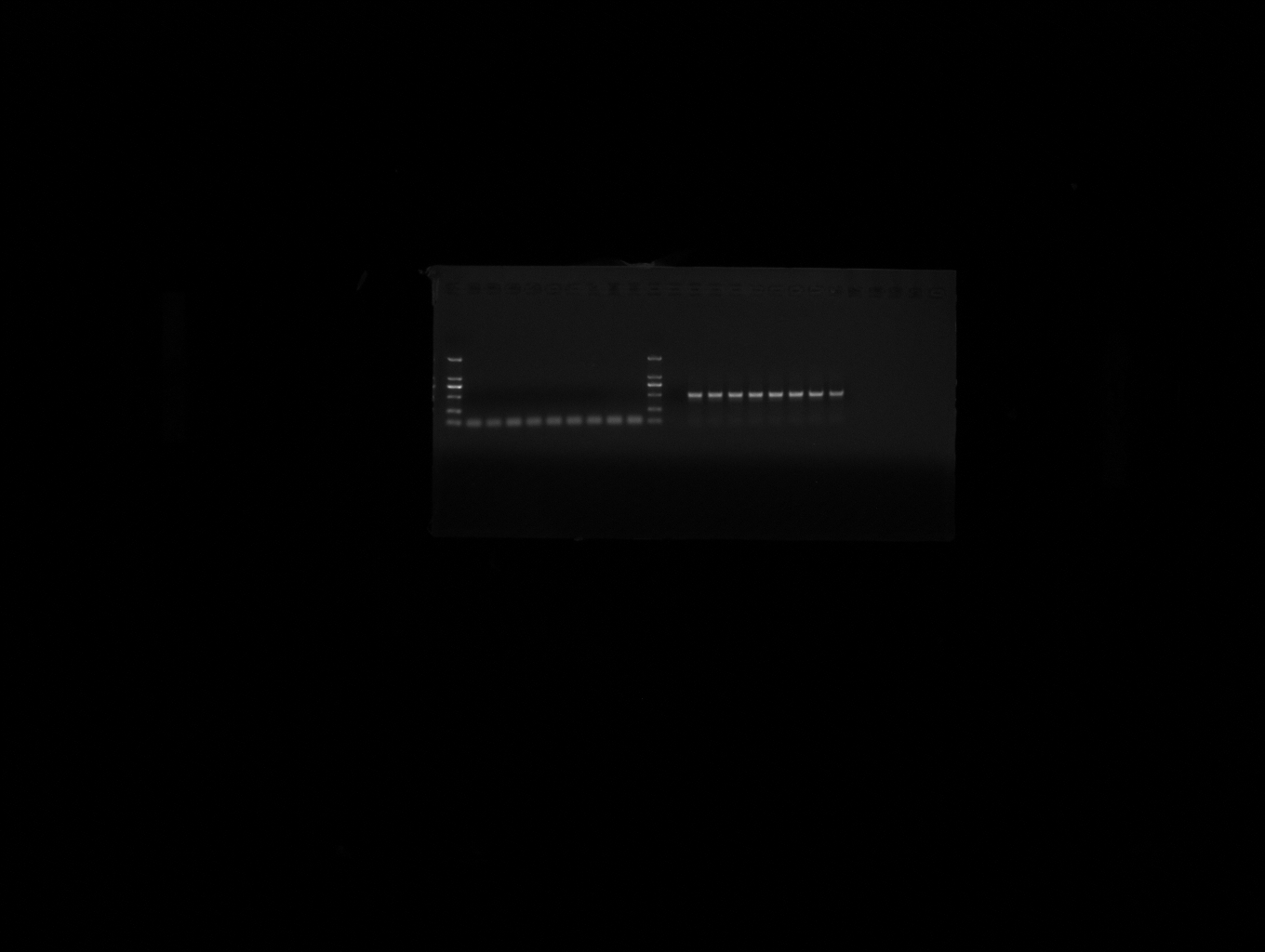

Supplement: Supplementary file 1 [file ijms-22-00084-s001.zip › ijms-1047367-for proofreading-supplementary/Supplemental information 1-3/Figure S3.tif]
